# A rare case of native mitral valve infective endocarditis by *Lactobacillus jensenii* in a healthy young patient

**DOI:** 10.1016/j.ijregi.2025.100648

**Published:** 2025-04-10

**Authors:** Thanaboon Yinadsawaphan, Narathorn Kulthamrongsri, Reed McCardell Malone, Salinda Surapongpairat, Chanokporn Puchongmart, Ben Thiravetyan, Korakrit Imwattana, Jutatip Na Witayanan

**Affiliations:** 1 Department of Medicine, John A. Burns School of Medicine, University of Hawai'i, Honolulu, Hawaii; 2 Department of Cardiovascular, Central Chest Institute of Thailand, Thailand; 3 Department of Surgery, Faculty of Medicine, Chulalongkorn University, Thailand; 4 Department of Internal Medicine, Texas Tech University Health Science Center, Lubbock, Texas; 5 Department of Microbiology, Faculty of Medicine Siriraj Hospital, Mahidol University, Thailand

**Keywords:** Lactobacillus jensenii, Lactobacillus, Endocarditis, Mitral valve, Myxomatous degeneration

## Abstract

*Lactobacillus jensenii*, a Gram-positive facultative anaerobic bacterium, is part of the normal vaginal microbiome and is occasionally found in fermented foods and probiotics. It is a rare cause of infections, with infective endocarditis (IE) being exceedingly uncommon. We report a case of IE caused by *Lactobacillus jensenii* in a previously healthy 15-year-old female who presented with progressive dyspnea, fever, and severe mitral regurgitation. Echocardiography revealed a vegetative mass on the anterior mitral leaflet. Blood cultures identified *L. jensenii* using Matrix-Assisted Laser Desorption/Ionization Time-of-Flight mass spectrometry. Surgical repair confirmed myxomatous degeneration and chronic inflammation of the mitral valve. The patient fully recovered after surgery and a 6-week antibiotic course. A review of 12 reported cases of *L. jensenii* IE, including our case, shows a predominance in immunocompetent females, possibly due to its role as a vaginal commensal. Most cases involved native valves with underlying pathology facilitating bacterial colonization. An empirical regimen with ampicillin and gentamicin is effective against *L. jensenii*. A treatment duration of 4-6 weeks is sufficient, with favorable outcomes.

## Highlights

• *Lactobacillus jensenii* infective endocarditis often presents with subacute constitutional symptoms.
• *L. jensenii* can infect both native and prosthetic valves in immunocompetent patients.
• An empirical regimen with ampicillin and gentamicin is effective in *Lactobacillus* infective endocarditis.
• Vancomycin can be used in *L. jensenii*, unlike other *Lactobacillus* strains.

## Introduction

*Lactobacillus jensenii* is a Gram-positive, rod-shaped, facultative anaerobic bacteria, which is a part of normal flora and is one of the dominant *Lactobacillus* species in a healthy female reproductive tract [1]. *L. jensenii* is also found in fermented food products such as yogurt and probiotic supplements [2,3]. *L. jensenii* infection is exceedingly rare, with infective endocarditis (IE) caused by this species being particularly uncommon. There are occasional reports of *L. jensenii* endocarditis, mostly in patients with predisposing conditions [4,5]. We present a rare additional case of IE caused by *L. jensenii* in a young, healthy female. Furthermore, to the best of our knowledge, this is the first comprehensive literature review specifically focused on *L. jensenii* endocarditis, compiling and summarizing all previously reported cases. This review serves as a practical reference to inform the diagnosis and management of this rare but potentially serious pathology.

## History of present illness

A previously healthy 15-year-old Thai female experienced progressive dyspnea on exertion, prompting referral to a regional cardiac center. Symptoms began 2 months prior with shortness of breath during physical activities, accompanied by intermittent low-grade fever. She had regular menstruation and denied a history of prior heart disease, smoking, drinking, substance use, prior sexual intercourse, and vaginal symptoms or discharge. She did not have significant food habits. She consumed yogurt occasionally.

On initial evaluation, her vital signs included a temperature of 38.5°C, blood pressure of 114/59 mmHg, heart rate of 61 bpm, and respiratory rate of 22 breaths/min. Physical examination upon arrival revealed a pansystolic murmur at the apex and bilateral pitting edema. She had good dentition and a normal skin and nail examination. There were no clinical findings suggestive of septic emboli, such as skin lesions or focal neurological deficits; therefore, additional imaging studies for embolic localization were not performed.

Initial laboratory results showed leukocytosis (white blood cell count of 14870 cells/µl) with a predominance of neutrophils (neutrophil: 81%). The erythrocyte sedimentation rate was 59 mm/hour (normal 0-30) and high-sensitivity troponin-T was 31 pg/ml (normal 0-14). The comprehensive metabolic panel was unremarkable.

**Figure 1:**
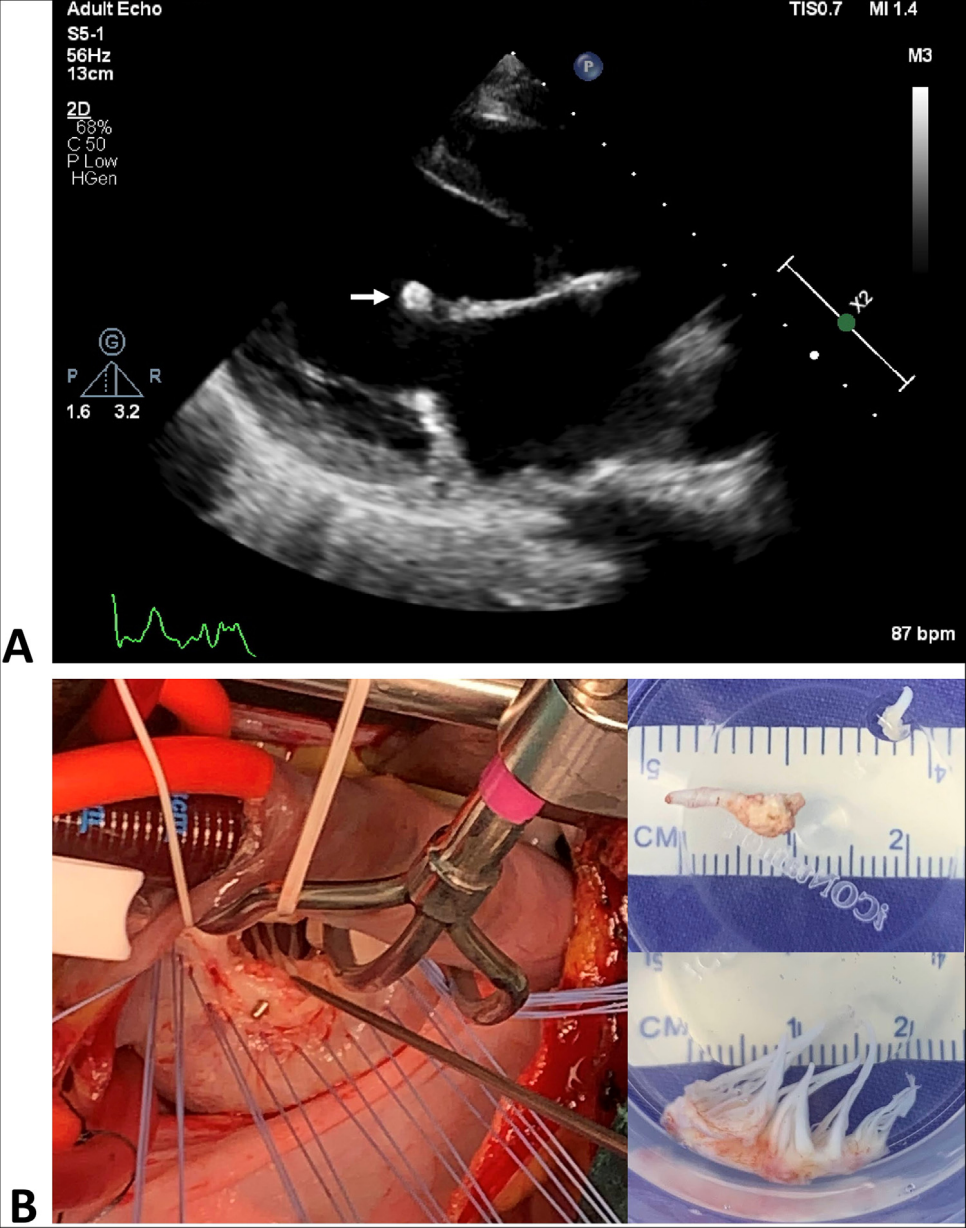
(a) A transthoracic echocardiogram showing a 0.9 cm x 0.7 cm oscillating mass on the anterior mitral leaflet (white arrow). (b) Intraoperative findings demonstrating the perforated P2 cusp of the mitral valve (left), two fragments of tissue from the mitral valve, measuring 1.2×0.5×0.2 cm on the fragment with vegetation (above), and 2.5×1.5×1.5 cm (below).

Initially, at a district secondary-care hospital, a transthoracic echocardiogram (TTE) revealed a frail anterior mitral leaflet with ruptured chordae tendineae, causing severe mitral valve regurgitation (MR). Repeated TTE showed severe MR with a 0.9×0.7 cm oscillating mass on the anterior mitral leaflet as shown in Figure 1. Ventricular function and the other valves were unremarkable.

Infective endocarditis was suspected and three blood cultures were drawn. All blood cultures grew *L. jensenii* after 4 days of inoculation. The blood culture specimens were additionally sent to a quaternary referral center for further identification. *L. jensenii* was confirmed by a Matrix-Assisted Laser Desorption/Ionization Time-of-Flight mass spectrometer (Bruker MALDI Biotyper® In Vitro Diagnostic Identification) with a confidence score value of 2.30 (High-confidence identification) and category A (high consistency).

The patient was initially treated with a diuretic and an empirical antibiotic regimen according to the 2023 ESC Guidelines for the management of IE, consisting of intravenous ampicillin (3 g every 6 hours), cloxacillin (3 g every 6 hours), and gentamicin (120 mg [3 mg/kg] once daily) [6]. This regimen was continued for 11 days until a mitral valve repair using a 28 mm cosgrove-edwards annuloplasty system.

Intraoperative findings demonstrated ruptured chordae tendineae with vegetation at the A2 and A3 MV cusps. The P2 cusp was perforated and grossly inflamed, slightly thickened, fibrotic, and calcified. Chordae tendineae were slightly thickened and fused. The histologic section revealed myxomatous mitral valve degeneration with evidence of mild chronic inflammation characterized by infiltration of lymphocytes, along with fibrosis and calcification, consistent with chronic change in IE. However, no organisms were identified within the valvular tissue histologic sections, possibly due to prior antibiotic therapy.

Post-surgery, antibiotics were changed to intravenous ceftriaxone (2 g once daily) and vancomycin (1500 mg daily; targeting a pre-dose trough level of 10-15 mg/l) for ease of administration, and warfarin was initiated. The patient was asymptomatic, and she was referred to the district secondary-care hospital for rehabilitation and a 6-week antibiotic course. At 3 months post-surgery, the patient was asymptomatic and had regained her previous functional status. Following discharge, the patient was referred to gynecology, where a work-up including a transabdominal ultrasound and pelvic examination was performed, revealing no abnormalities. No further gynecological follow-up was recommended.

## Discussion

*L. jensenii* is one of the dominant species in the female lower reproductive tract, accounting for 22% of the healthy vaginal microbiome [7]. *Lactobacillus* colonization protects against sexually transmitted diseases, bacterial vaginosis, and vaginal candidiasis [8].

*Lactobacillus* is rarely pathogenic but occasionally causes infections, particularly in patients who have predisposing conditions. IE caused by *Lactobacillus* species is extremely rare. A recent literature review showed that 82 *Lactobacillus* IE patients were reported on databases until September 2023 [9]. Of those, only 11 cases (13.4%) were affected by *L. jensenii*. We further identified 11 reported cases of *L. jensenii* IE by searching PubMed using the search terms (“Lactobacillus” AND “endocarditis”). The 11 reported *L. jensenii* IE cases and our case was reviewed as shown in Supplement Table 1. *L. jensenii* IE patients usually presented with constitutional symptoms, including subacute onset low-grade fever, malaise, night sweats, generalized weakness, and occasionally heart failure symptoms depending on valve damage severity.

Of the 12 patients with *L. jensenii* IE, nine patients (75%) were female, as opposed to male predominance as reported in Ioannou *et al.*’s narrative review [9]. In said review, males accounted for 69% (55/79) of all IE cases caused by *Lactobacillus spp.* and males greatly predominated among cases caused by every organism *except L. jensenii*. Since *L. jensenii* is an inhabitant in a female reproductive tract, females might be exposed to this *Lactobacillus* species more often than males. Indeed, other reported IE-causing organisms (*L. rhamnosus, casei, paracasei, plantarum,* etc) are not major vaginal commensals [10] and their infections are skewed toward male patients.

*L. jensenii* IE can affect both native valves and prosthetic heart valves. Most of the reported cases were patients with native valves; there was only one case with a valve prosthesis and one with a previously repaired mitral valve. However, normal valvular endothelium is resistant to bacterial colonization. Alteration of a cardiac valve surface due to certain underlying valvular heart pathology to create a proper site for bacterial attachment is a prerequisite condition for IE development [11]. Although our patient was previously healthy, histopathology of the affected valve displayed myxomatous degeneration of the mitral valve, which was a histologic characteristic of degenerative mitral valve disease similar to Bepna’s patient [5]. In addition to degenerative mitral valve disease, another significant cardiac comorbidity observed in *L. jensenii* IE cases is the bicuspid aortic valve. Non-cardiac risk factors associated with these cases included neurogenic bladder, obstructed ureteric calculi, and recent obstetric procedures. A definitive bacterial entry point was not clearly identified in most previously reported cases, although poor dentition was commonly observed. However, establishing this as the definite source of bacterial entry remains challenging and represents a limitation of the current literature. Immunocompromised status is not required for *Lactobacillus* endocarditis. Only 7.3% of the IE patients caused by *Lactobacillus* species had immunosuppression status [9], and none of the reported *L. jensenii* IE patients were immunocompromised.

Generally, *Lactobacillus* species are susceptible to penicillin, ampicillin, erythromycin, clindamycin, tetracycline, quinupristin-dalfopristin, and linezolid [12,13]. Most *Lactobacillus* species have intrinsic resistance to vancomycin and most aminoglycosides except gentamicin. Interestingly, all species in *L. delbrueckii* phylogroup including *L. jensenii* are susceptible to vancomycin as a result of the substitution of tyrosine to phenylalanine on an active site of D-Ala-D-Ala ligase [12]. Of the review of the 12 *L. jensenii* IE cases, there is no report of vancomycin resistance on drug susceptibility testing. *Lactobacillus* species display high resistance to most aminoglycosides due to lack of cytochrome-mediated drug transport [13]. In contrast to other aminoglycosides, gentamicin has a superior ability to cross a cell membrane, contributing to a high-level susceptibility for *Lactobacillus* species [13].

For community-acquired native valve IE, the current recommended empirical regimen including ampicillin in combination with ceftriaxone, cloxacillin, and gentamicin, seems to cover *Lactobacillus* species [6]. Based on case reports [14,15] and in vitro evidence [12], a combination of ampicillin and gentamicin is a reasonable choice of treatment for *Lactobacillus* IE. Vancomycin may be used only against *L. delbrueckii* phylogroup including *L. jensenii* [12]. Since systematic assessment of antimicrobial duration in *Lactobacillus* IE is limited, the duration of therapy reconciles standard guidelines and the specific affected valves. Of those 12 *L. jensenii* IE cases, 4-6 weeks of the antimicrobial combination was sufficient. There is no report on therapeutic failure, recurrent infection with the same organism, or death among those 12 *L. jensenii* IE cases. However, most previous reports did not specify the duration of follow-up after treatment, limiting the assessment of long-term outcomes.

## Conclusion

In conclusion, *L. jensenii* endocarditis is a rare infection, with a limited number of cases reported in the literature. *L. jensenii* is a major vaginal commensal and can be found in fermented food products and probiotic supplements. Consequently, *L. jensenii* IE can cause both native and prosthetic heart valves regardless of immune status, albeit rarely.

Managing *L. jensenii* endocarditis is challenging due to limited data. However, based on available evidence, a combination of ampicillin and gentamicin is an effective empirical regimen for treating *Lactobacillus* IE, followed by a step-down regimen per drug sensitivity. Vancomycin can be used in *L. jensenii,* unlike other *Lactobacillus* strains. A treatment duration of 4-6 weeks appears effective, with favorable outcomes observed in reported cases. Further research is still needed to assess treatment regimens.

## Declarations of competing interest

The authors have no competing interests to declare.

## Appendix Supplementary materials

Image, application 1
