# Supplementary material for: A rare case of native mitral valve infective endocarditis by Lactobacillus jensenii in a healthy young patient
Source: IJID Reg. 2025 Apr 10;15:100648. doi: 10.1016/j.ijregi.2025.100648 (PMC12225030; doi:10.1016/j.ijregi.2025.100648)
Supplement: Supplementary file 1 [file mmc1.docx]

Supplement table 1:
Summary of previously published *Lactobacillus jensenii* infective endocarditis cases and our reported case.

| Author, year | Age and gender | Onset and Symptoms | Comorbidities or predisposing factors | Affected valve | Sources of *L. jensenii* isolation | Antibiotics and duration | Recurrent or death |
| --- | --- | --- | --- | --- | --- | --- | --- |
| Atkins,  1990[1] | 61-year-old  male | Onset: 3 weeks  Intermittent fever  Night sweat  Malaise  Progressive dyspnea | Multiple previous episodes of IE with prosthetic aortic valve replacement  Severe periodontal disease  Multiple colonic polyp with carcinoma in situ | Prosthetic aortic valve | 4 sets of blood cultures (8 total blood culture bottles) | Ampicillin + netilmicin for 6 weeks | No |
| Puleo,  1994[2] | 21-year-old  female | Onset: 1 week  Fever  Dry cough | Supracristal ventricular septal defect  Multiple dental caries | Native pulmonic valve | 4 out of 6 sets of blood cultures, anaerobic bottles | ampicillin + gentamicin for 1 week, following by penicillin and gentamicin for a total of 26 days | No |
| Vaghjimal,  1997[3] | 80-year-old  female | Onset: unknown  Fever  Shortness of breath | Hypertension  Dementia  Atrial fibrillation  Poor dentition | Native aortic valve | 2 sets of blood cultures | Ampicillin + gentamicin for 4 weeks | No |
| Khan,  2005[4] | 16-year-old  female | Onset: Acute  Low grade fever  Left side chest pain due to antero-apical wall MI from septic embolization of mid-distal LAD | None | Native mitral valve | 2 sets of blood cultures | Ampicillin + gentamicin for 6 weeks | No |
| Fradiani,  2010[5] | 47-year-old  female | Onset: 10 days  Fever  Generalized weakness  Pharyngalgia | Hypertension | Native mitral valve | 3 sets of blood cultures, anaerobic bottles  Resected mitral valve (identified by 16S rRNA gene sequencing) | Amoxicillin-clavulanic acid + amikacin,  following by teicoplanin + meropenem for another 4 weeks | No |
| Suarez-Garcia, 2012[6] | 27-year-old  female | Onset: 20 days  Fever  Lower extremity pain  Petechiae of both feet | Recent elective abortion by dilatation and curettage | Native aortic valve | 2 sets of blood cultures, aerobic bottles | Penicillin + gentamycin for 6 weeks | No |
| Marciniak,  2014[7] | 31-year-old  female | Onset: 1 month  Fever  Myalgia  Malaise  Lethargy | Recent vaginal delivery  Bicuspid aortic valve | Native bicuspid aortic valve | 2 sets of blood cultures | Teicoplanin + rifampicin for 10 weeks | No |
| Patnaik,  2015[8] | 56-year-old  male | Onset: 3 days  Generalized weakness  Chest pain  Shortness of breath | Diabetes  Neurogenic bladder with chronic indwelling urinary catheterization.  Poor dentition | Native aortic valve with aortic root abscess | Multiple blood cultures, anaerobic bottles (number of samples unspecified)  Resected aortic valve | Vancomycin + piperacillin–tazobactam,  following by ampicillin/sulbactam,  following by penicillin for a total of 6 week | No |
| Minto,  2020[9] | 57-year-old  female | Onset: 6 weeks  Cough  Chills  Shortness of breath | Hypertension  Diabetes  Asymptomatic bilateral obstructing ureteric calculi | Native aortic valve | Blood cultures (number of samples unspecified) | Amoxicillin + clarithromycin  (Not mention duration) | No |
| Grazioli-Gauthier,  2022[10] | 40-year-old  male | Onset: 1 month  Fatigue  Vomiting  Diarrhea  Weight loss | Previous mitral valve repair due to a congenital anterior leaflet prolapse  Epilepsy | Repaired mitral valve | 3 sets of blood cultures  Resected mitral valve  Thrombus culture | Amoxicillin + gentamycin for 1 week, following by amoxicillin alone for a total of 6 weeks | No |
| Bapna,  2023[11] | 22-year-old  female | Onset: 1 month  Night sweat  Chills  Chest tightness  Palpitation  Shortness of breath | Myxomatous anterior mitral leaflet  Ligation of a patent ductus arteriosus | Myxomatous mitral valve. | 6 sets of blood cultures  Resected mitral valve | Meropenem + vancomycin for 10 days,  following by ertapenem for a total of 6 weeks | No |
| Yinadsawaphan, (Our case) | 15-year-old  female | Onset: 2 months  Fever  Chest tightness  Shortness of breath | Myxomatous mitral valve degeneration | Myxomatous mitral valve | 3 sets of blood cultures | Ampicillin + cloxacillin + gentamycin for 11 days, following by ceftriaxone + vancomycin for 6 weeks | No |

Supplement reference

[1] Atkins MC, Nicolson L, Harrison GA, Paull A, Malnick H, Morrison D. Lactobacillus jensenii prosthetic valve endocarditis*.* *J Infect* 1990, 21:322-324, doi:10.1016/0163-4453(90)94245-u.

[2] Puleo JA, Shammas NW, Kelly P, Allen M. Lactobacillus isolated pulmonic valve endocarditis with ventricular septal defect detected by transesophageal echocardiography*.* *Am Heart J* 1994, 128:1248-1250, doi:10.1016/0002-8703(94)90761-7.

[3] Vaghjimal A, Lutwick LI, Chapnick EK. Endocarditis caused by Lactobacillus*.* *Postgrad Med J* 1997, 73:61-62, doi:10.1136/pgmj.73.855.61-a.

[4] Khan F, Khakoo R, Failinger C. Managing embolic myocardial infarction in infective endocarditis: current options*.* *J Infect* 2005, 51:e101-105, doi:10.1016/j.jinf.2004.10.006.

[5] Fradiani PA, Petrucca A, Ascenzioni F, Di Nucci G, Teggi A, Bilancini S, Cipriani P. Endocarditis caused by Lactobacillus jensenii in an immunocompetent patient*.* *J Med Microbiol* 2010, 59:607-609, doi:10.1099/jmm.0.017764-0.

[6] Suarez-Garcia I, Sanchez-Garcia A, Soler L, Malmierca E, Gomez-Cerezo J. Lactobacillus jensenii bacteremia and endocarditis after dilatation and curettage: case report and literature review*.* *Infection* 2012, 40:219-222, doi:10.1007/s15010-011-0182-9.

[7] Marciniak A, Karapanagiotidis GT, Sarsam M, Sharma R. Postpartum Lactobacillus jensenii endocarditis in patient with bicuspid aortic valve*.* *J Thorac Cardiovasc Surg* 2014, 148:e219-221, doi:10.1016/j.jtcvs.2014.05.096.

[8] Patnaik S, Davila CD, Chennupati A, Rubin A. Endocarditis of the native aortic valve caused by Lactobacillus jensenii*.* *BMJ Case Rep* 2015, 2015, doi:10.1136/bcr-2014-206288.

[9] Minto T, Bullock N, Deglurkar I, Hughes O. Asymptomatic bilateral obstructing ureteric calculi resulting in Lactobacillaemia and endocarditis requiring emergency aortic valve replacement*.* *Urol Case Rep* 2020, 32:101218, doi:10.1016/j.eucr.2020.101218.

[10] Grazioli-Gauthier L, Rigamonti E, Leo LA, Martinetti Lucchini G, Lo Priore E, Bernasconi E. Lactobacillus jensenii mitral valve endocarditis: Case report, literature review and new perspectives*.* *IDCases* 2022, 27:e01401, doi:10.1016/j.idcr.2022.e01401.

[11] Bapna M, Maurer J, Ruddy S, Karnik K, Turett G, Urban C, Yoon J, Prasad N, Yung L, Lang S, et al. A case of Lactobacillus jensenii associated native valve endocarditis*.* *IDCases* 2023, 32:e01806, doi:10.1016/j.idcr.2023.e01806.
